# Unleashing the prognostic value of atrial shape in atrial fibrillation

**DOI:** 10.1016/j.hroo.2021.11.011

**Published:** 2021-11-19

**Authors:** Pablo Lamata

**Affiliations:** School of Biomedical Engineering and Imaging Sciences, King’s College London, London, United Kingdom

The heart remodels when exposed to changes in demand or to an insult. The myocardial walls thicken in response to heavier workloads, and the heart experiences eccentric remodeling in the late stages of heart failure. Changes in cardiac morphology are useful objective signs to detect and monitor the progression of several cardiovascular diseases, and to optimally plan their therapy. In this context, recent progresses in medical imaging and computational anatomy now allow for unprecedented detail in the analysis of cardiac remodeling^1^. Cardiac anatomy is more than a simple volume, mass, or thickness, and current technology allows to explore much more subtle changes in the 3-dimensional (3D) shape of the cardiac chambers. The quest is thus to find those anatomical traits that have specific diagnostic or prognostic value.

It is well known that atrial fibrillation (AF) begets AF: the loss of the healthy activation patterns triggers a series of mechanisms that leads to microstructural adverse remodeling that will further contribute to the exacerbation of this condition. AF will then induce 3D changes in the shape of the atrium, increasing its volume, the anterior-to-posterior (AP) diameter, and its sphericity. There are thus clear micro and macro signs of atrial remodeling that inform about the progression of the condition. But one of the intriguing questions towards an improved management of AF and the decision-making is: What are the changes in the 3D shape of the left atrium (LA) that could predict the success of the ablation procedure?

Several research teams have tackled this question, and there is a general agreement in the idea that the predictive changes lay in the metrics that inform of the AF burden, ie, LA volume and dilation in the AP direction. An early work in the computational anatomical study of the LA^2^ neatly described how the dilation of the LA in the AP direction was the main characteristic that progressively described the differences between controls, paroxysmal AF, and persistent AF. This study also provided the interesting finding that the AP dilation was not correlated with an increase of volume within the paroxysmal and persistent AF subgroups. This is a piece of evidence of the existence of different remodeling patterns that respond to the AF burden with independent amounts of AP dilation and LA volume expansion.

The LA sphericity is the metric that seems to capture the relevant relationship between AP distance and LA volume, as it has been shown to outperform them in the prediction of AF recurrence (odds ratio of 1.36 vs 1.16 for diameter and 1.024 for volume).^3^ The beauty in the metrics of AP diameter, LA volume, and sphericity is in their simplicity. Besides, thinking on the mechanisms of reentry, the size of the substrate is a clear explanation—the larger the LA, the larger the chances to find the repolarized region to reactivate. And in general, any metric that shows a correlation with the AF burden should be a sign of the microstructural remodeling, and the later we get into the intervention, the smaller the chances of success. But one could quickly think that these bulk shape descriptors might not be so specific to the question asked: What is in the shape of the LA that specifically predisposes to a failure in the ablation procedure?

The general agreement across different studies is that there is something in the asymmetry of the LA, but there is a lack of consensus on its definition and findings across different cohorts. One proposal is the anterior asymmetry where a specific volumetric expansion in the anterior part of the LA is associated with an increased recurrence,^4^ and another is the vertical asymmetry where the feature that favors the recurrence is the expansion in the roof of the LA.^5^ In this issue of *Heart Rhythm O*^*2*^, Jia and colleagues^6^ have now provided further insights, bringing a neat and comprehensive experimental design: correcting for volume changes and AF persistence, they have found the anatomical feature with an outstanding predictive value (odds ratio of 6.2) that relates to the anatomy of the roof of the atrium and orientation of the pulmonary veins.

This finding might then be the keystone we were missing to inform clinical decisions; it does seem to be the anatomical trait that predicts risk of recurrence. But the quest has not finished here; there are 2 key steps missing—the reproduction of this performance in an external independent cohort, and the mechanistic explanation of the finding: Why are those changes depicted in Figure 6^6^ predictive of AF recurrence? The initial hunch does indeed point to the study of the interplay between the atrial body and the pulmonary veins, where the ablation takes place. And once this mechanism is revealed, what will then be the best metric to characterize it? Could it be a simple V shape vs flat-coved shape of the LA roof,^7^ as seen during the ablation? The quest continues, and the use of computational models of cardiac physiology does indeed provide a neat methodology to study the interplay between the shape and the function.^8^

## Funding Sources

The author holds a Wellcome Trust Senior Research Fellowship (209450/Z/17/Z).

## Disclosures

The author is a researcher whose strategic interest is to see the use of computational anatomical tools translated into clinical practice. Not related to this work, the author also sits at the Scientific Advisory Board of Ultromics Ltd and is a shareholder in Congenita Ltd.

## References

[1] Fonseca C.G., Backhaus M., Bluemke D.A. (2011). The Cardiac Atlas Project--an imaging database for computational modeling and statistical atlases of the heart. Bioinformatics.

[2] Cates J., Bieging E., Morris A. (2015). Computational shape models characterize shape change of the left atrium in atrial fibrillation. Clin Med Insights Cardiol.

[3] Bisbal F., Guiu E., Calvo N. (2013). Left atrial sphericity: a new method to assess atrial remodeling. Impact on the outcome of atrial fibrillation ablation. J Cardiovasc Electrophysiol.

[4] Nedios S., Tang M., Roser M. (2011). Characteristic changes of volume and three-dimensional structure of the left atrium in different forms of atrial fibrillation: predictive value after ablative treatment. J Interv Card Electrophysiol.

[5] Varela M., Bisbal F., Zacur E. (2017). Novel computational analysis of left atrial anatomy improves prediction of atrial fibrillation recurrence after ablation. Front Physiol.

[6] Jia S., Nivet H., Harrison J. (2021). Left atrial shape is independent predictor of arrhythmia recurrence after catheter ablation for atrial fibrillation: a shape statistics study. Heart Rhythm O2.

[7] Kurotobi T., Iwakura K., Inoue K. (2011). The significance of the shape of the left atrial roof as a novel index for determining the electrophysiological and structural characteristics in patients with atrial fibrillation. Europace.

[8] Corral-Acero J., Margara F., Marciniak M. (2020). The “Digital Twin” to enable the vision of precision cardiology. Eur Heart J.

